# Seed Treatments for Management of Soybean Cyst Nematode, *Heterodera glycines*, in Mid-Atlantic Soybean Production

**DOI:** 10.2478/jofnem-2023-0026

**Published:** 2023-08-01

**Authors:** Alexandra C. Kessler, Alyssa M. Koehler

**Affiliations:** Department of Plant and Soil Sciences, University of Delaware, Newark, DE 19716

**Keywords:** biological control, *Heterodera glycines*, seed treatment, soybean cyst nematode, soybean management

## Abstract

Soybean Cyst Nematode (SCN), *Heterodera glycines* Ichinohe, is the most important pathogen of soybean in the Mid-Atlantic region. In recent decades, a decline in the effectiveness of genetic resistance has been observed and additional management approaches are needed. Seed treatments are of rising interest, but no local data on product response exists for the region. In 2020–2021, two experiments were conducted to observe the effects of chemical and biological seed treatment options. In one experiment, chemical seed treatments pydiflumetofen (Saltro®) and fluopyram (ILEVO®) were screened against nontreated plain seed for SCN suppression. In a second experiment, pydiflumetofen, fluopyram and four biological nematode-protectant seed treatments with a standard base insecticide and fungicide treatment were compared to nontreated plain seed and seed with only the standard base treatment to test product efficacy against SCN. Seed treatments increased the percent emergence over plain seed. Nematode reproductive factors and female counts from roots were collected, but did not statistically differ between seed treatments or plain seed. Yield differences were observed in one of the five trials, where pydiflumetofen + base seed treatment yielded the highest (*p* < 0.001) at 3813.1 kg/ha. Response from seed treatments varied, with no specific seed treatment consistently reducing SCN populations or increasing yield across trials. Seed treatments may have potential as an element of an integrated management approach for SCN.

In 2019, United States (U.S.) soybean (*Glycine max* [L.] Merrill) production exceeded 97,000 MT (3,552,000 bu) covering roughly 31 million hectares (USDA ERS, 2022). Soybeans are also an important crop within the Mid-Atlantic region and were the second crop grown by acre in Delaware (DE) and Maryland (MD) in 2019, accounting for over 60 million dollars of revenue (USDA ERS, 2022). Soybean cyst nematode (SCN), *Heterodera glycines* Ichinohe, is the most yield-limiting pest of soybean within the Mid-Atlantic (Allen et al., 2017; Bandara et al., 2020). Sandy soils in DE and Eastern Shore MD favor the proliferation of SCN, with yield loss estimates exceeding 7,076 MT (260,000 bu) across both states annually (Allen et al., 2021).

Hatched SCN juveniles stage 2 (J2) penetrate and embed themselves within the plant roots, where they establish a feeding site and consume cell contents to survive (Davis and Tylka, 2021). Females break through the root surface, remaining attached, and swell full of eggs. A single female can fill with over 250 eggs (Lauritis et al., 1983). Symptoms of SCN infection include stunting, yellowing of the plant, and plant death. Even in the absence of aboveground symptoms, SCN can reduce yields up to 30% (Noel, 1992; Niblack, 2005). As females die, their body walls harden, leaving a cyst around the eggs. This cyst provides a survival structure for the eggs after it is dislodged from the roots into the soil (Duan et al., 2009). Eventually, the cyst will burst, and the eggs will be released, continuing the cycle (Davis and Tylka, 2021). SCN are often found in “hot spots” throughout a field and can be spread by mechanical equipment or rainwater runoff (Davis and Tylka, 2021). The ability of cysts to persist for long periods limits the effectiveness of cultural management approaches such as short crop rotations with nonhost crops such as maize (*Zeae mays* L.) (Koenning, 2004).

In addition to cultural practices, SCN management includes genetic, chemical, and biological approaches. For decades, host resistance was the primary tool for SCN management. Plant Introduction 88788 (PI 88788) is the most common source of genetic resistance (Niblack et al., 2008). Nationally, there are seven main breeding lines, but since 1990, most resistant cultivars have been developed using PI88788 (Davis and Tylka, 2021). When first released, PI88788 kept SCN reproduction rates below 10%. Observations of increased SCN reproduction began in the midwest in the early 2000s, and nearly all surveys in that region over the past two decades have indicated increasing virulence of SCN and decreasing yield in soybean varieties relying on PI88788 (McCarville et al., 2017). This same observation was made during a 2009–2010 survey in DE where a shift in population was observed and reproduction levels on resistance gene PI88788 averaged 67% (Mulrooney and Gregory, 2010).

Fumigants, such as Telone C-35 and chloropicrin, when used in combination with other controls, have been shown to increase yield in SCN-infested fields (De Bruin and Pedersen, 2008). Soil-applied nematicides may result in larger populations at the end of the season than at application due to the short window of efficacy and resurgence of population once root protection declines (Wrather et al., 1984; Davis and Tylka, 2021). Over time, the number of fumigant options have decreased due to health and environmental concerns. Additionally, traditional granular and soil-applied fumigant nematicides are typically cost prohibitive for large-scale use in soybeans (Zasada et al., 2010; Jensen et al., 2018). With decline in effectiveness of resistance genes and fumigant nematicide options, additional SCN management tools are needed.

Soybean seed receiving at least one seed treatment product rose from 8% to 75% over the years of 1996 to 2013 in the U.S. (Munkvold et al., 2014). Treatment of seed allows for application of multiple products such as fungicides, insecticides, and/or nematode-protectants. Nematode-protectant seed treatments may include chemical or biological control agents with varying modes of action. Two recently launched chemical seed treatments, Saltro (pydiflumetofen, succinate dehydrogenase inhibitor (SDHI), Syngenta, Basel, Switzerland) and ILEVO (fluopyram, SDHI, developed by BASF, Ludwigshafen, Germany), are fungicides with nematistatic activity. Fluopyram was registered for soybean seed treatment use in 2014 (US EPA, 2014) followed by pydiflumetofen in 2019 (US EPA, 2019). The SDHI class of fungicides inhibit mitochondrial respiration in fungi and both products are also labeled for control of *Fusarium virguliforme*, the causal agent of soybean sudden death syndrome (SDS) and *Septoria glycines*, causal agent of Septoria brown spot. Symptoms of SDS often appear earlier in the season and at greater severity in fields with SCN (McLean et al., 1993). In controlled environments, fluopyram has been shown to reduce SCN hatching, juvenile movement, and ability of juvenile SCN to penetrate soybean roots (Xing and Westphal 2006; Beeman and Tylka, 2018). In greenhouse conditions, pydiflumetofen has also been shown to reduce egg hatching and number of SCN cysts per plant (Dhital, 2020). Bissonnette et al. (2020) assessed fluopyram-treated seed for impact on SCN population density in fields with low SDS index levels throughout Iowa. No seed treatment trials have been conducted for fluopyram or pydiflumetofen in DE. In recent years, several biological agents have also come on the market for application as soybean seed treatments, with no local data for the Mid-Atlantic. The objectives of this study were to 1) compare performance of two chemical seed treatments recently labeled for management of SCN in soybean, and 2) assess performance of biological seed treatments compared to chemical standards for management of SCN in Mid-Atlantic soybean production.

## Materials and Methods

### Experimental Design

From 2020–2021, two small-plot experiments were conducted to assess the impact of nematode-protectant seed treatments on SCN population density and end of season yield. The first experiment was established in a field with pepperbox loamy sand (80–98% sand) and known SCN pressure in Georgetown, DE. Soybeans were planted in the two seasons prior (2018–2019), and glyphosate herbicide was used for weed control, no foliar applications of fungicide or insecticide were made. In 2021, an additional site with low SCN pressure was added at the Wye Research and Education Center in Queenstown, MD at a field with Nassawango silt loam soil type (20–50% sand) that had been previously planted in corn (Table 1). The soybean cultivar GH3934X (Syngenta) was planted at 272,000 sd/ha (110,000 sd/A) on April 19, 2020, and April 27, 2021, in Georgetown and June 4, 2021, in Queenstown. The Georgetown trials were planted using a Kinze (Kinze, Williamsburg, IA) plot planter equipped with an Almaco cone-type seeder (Almaco, Nevada, Iowa) with four row plots 8.8 m in length on a 0.76 m spacing in a randomized complete block design with five replications. The Queenstown trial was planted using a Great Plains EWNT-10 with six row plots 8.8 m in length on a 76.2 cm spacing in a randomized complete block design with five replications. Treatments included fluopyram (ILEVO®, 0.15 mg a/seed, BASF), pydiflumetofen (Saltro®, 0.075 mg a/seed, Syngenta), and a nontreated plain seed control for a total of 15 plots in each trial (Table 2). The second experiment was conducted from 2020–2021 in the same field in Georgetown, DE (Table 1). The soybean cultivar 4444RXS (Progeny) was planted at 346,000 sd/ha (110,000 sd/A) on April 19, 2020, and April 27, 2021, in four row plots 8.8 m in length on a 0.76 m spacing with six replications. Treatments included a nontreated plain seed control, seed with a base treatment [Allegiance (Metalaxyl, 4 g a/100kg, Bayer Crop Science), Stamina (pyraclostrobin, 7.5 g a/100 kg, BASF), Systiva XS Xemium Brand (fluxapyroxad, 5 g a/100 kg, BASF), Poncho 600 (clothianidin, 0.11 mg a/seed, BASF), Flo Rite (66 ml/100 kg, BASF), and Color Coat Red (33 ml/100 kg, BASF)], and seed with the base treatment and one of six nematode-protectant products for a total of 48 plots in each trial. Nematode-protectant treatments included fluopyram (ILEVO®, 0.15 mg a/seed, BASF), pydiflumetofen (Saltro^®^, 0.075 mg a/seed, Syngenta), heat-killed *Burkholderia rinojenses* and its spent fermentation media (BioST^®^Nematicide 100, 195 ml/100 kg, Albaugh), *Bacillus amyloliquefaciens* strain PTA-4838 (Aveo™EZ, 2ml/100,000 seed, Valent), *Pasteuria nishizawae* (Clariva^®^pn, 130 ml/100 kg seed, Syngenta), and *Bacillus amyloliquefaciens* strain MBI 600 and cis-Jasmone (Trunemco™, 20.2 ml/100 kg seed, Nufarm) (Table 2). Both soybean cultivars contained the PI88788 source of resistance, and seed treatments were applied by the manufacturer. In all experiments, the center two rows of each plot were harvested mechanically with a plot combine. A Massey Ferguson 8XP plot combine (AGCO, Duluth, GA) was used at the Georgetown fields and an Almaco R1 was used in Queenstown. Grain yield and moisture were collected, and grain yield was adjusted to 13% moisture. Weather data for these experiments was collected using the Delaware Environmental Observing System (DEOS), Georgetown-REC station in Georgetown, DE.

**Table 1.** Locations, crop information, SCN sampling dates, and initial SCN counts for each field site in this study.

**Year**	**Location**	**Experiment**	**Planting Date**	**Harvest Date**	**Previous Crop**	**Irrigation^z^**	**Sampling Date for SCN**		**Date of SCN Female Count**	**Initial SCN Count^y^**

							**At Planting**	**End of Season**		
2020	Georgetown, DE	1	19 Apr	22 Oct	Soybean	Yes	19 Apr	14 Sept	16 Jun	936
2021	Georgetown, DE	1	27 Apr	5 Nov	Soybean	Yes	27 Apr	27 Aug	3 Jun	61
2021	Queenstown, MD	1	4 Jun	12 Nov	Corn	No	18 May	1 Sept	7 Jul	31
2020	Georgetown, DE	2	19 Apr	22 Oct	Soybean	Yes	19 Apr	6 Oct	15 Jun	34263
2021	Georgetown, DE	2	27 Apr	5 Nov	Soybean	Yes	27 Apr	13 Sept	3 Jun	3917

z Water was applied through overhead irrigation during the summer months to supplement natural rainfall.

y Initial soybean cyst nematode (SCN) counts in spring prior to or at the time of planting. Experiment 1 is the average number of SCN J2 and cysts/500 cm^3^ soil across all plots for each year and Experiment 2 is the average number of SCN eggs/250 cm^3^ soil across all plots for each year.

**Table 2.** Seed treatments used in field trials from 2020–2021.

**Treatment Name**	**Product Name**	**Active Ingredient (AI)**	**Application Rate**	**Mode of Action**	**Group**
Nontreated^1,2^	n/a	n/a	n/a	n/a	n/a
Fungicide + Insecticide base^2^	Allegiance FL Stamina Systiva XS Xemium Poncho 600	Metalaxyl Pyraclostrobin Fluxapyroxad Clothianidin	4 g a/100 kg 7.5 g a/100 kg 5 g a/100 kg 0.11 mg a/seed	PA QoI SDHI nAChR	Group 4 Group 11 Group 7 Group 4(A)
Saltro^1^ + base^2^	Saltro®	Pydiflumetofan	0.075 mg a/seed	SDHI	–
ILEVO^1^ + base^2^	ILEVO®	Fluopyram	0.15 mg a/seed	SDHI	N-3
Clariva + base^2^	Clariva^®^pn	*P. nishizawae*	130 ml/100 kg	Biologic	N-UNB
Trunemco + base^2^	Trunemco™	*B. amyloliquefaciens* cis–jasmone	20.2 ml/100 kg	Biologic	N-UNB
Aveo EZ + base^2^	Aveo™ EZ	*B. amyloliquefaciens* strain PTA-4838	2 ml/100,000 seeds	Biologic	N-UNB
BioST + base^2^	BioST^®^Nematicide100	*Burkholderia rinojenses*	195 ml/100 kg	Biologic	N-UNB

1 Indicates use in Experiment 1

2 Indicates use in Experiment 2

### Stand Counts

Stand counts were recorded at 14 and 28 days after planting (DAP) or emergence (DAE) in all trials. All emerged seedlings were counted in rows two and three of each plot. Average seedlings from the middle two rows were divided by the initial seeding rate to determine a percent emergence value.

### Quantifying SCN populations

Soil sampling was used to determine population levels in each treatment. Soil samples were collected immediately after planting to determine the initial population density [Pi] and at the end of the season prior to harvest for a final population density [Pf]. Soil cores were collected from the middle two rows of each plot by moving in a zig-zag pattern to obtain 20–30 soil cores from the base of soybean plants at a depth of 10–15 cm. Soil cores from each plot were mixed into a single plastic bag and stored at 18°C for up to five days until they were shipped for processing. Samples from Experiment 1 were submitted to the North Carolina Department of Agriculture and Consumer Services’ Nematode Assay Section, where 500 cm^3^ of soil from each plot was extracted using a combination of elutriation (Byrd et al., 1976) and sugar centrifugal flotation (Jenkins, 1964). Counts of cysts and J2 were determined. Samples from Experiment 2 were submitted to SCN Diagnostics at the University of Missouri. From each soil sample, SCN cysts were extracted from 250 cm^3^ of soil and collected on a 250 μm-pore sieve using modified wet-sieving and decanting methods (Gerdemann, 1955). Eggs were extracted from cysts with a motorized rubber stopper (Faghihi and Ferris, 2000), collected on a 25 μm-pore sieve, stained, and counted. The reproductive factor (Rf) was calculated by dividing the Pf by Pi to generate a ratio to measure the change in number of SCN per volume of soil over the entire growing season for each plot. When Rf=1, no change in SCN population density occurred in the season; if Rf <1, there was a decrease in SCN population density over the season; if Rf >1, there was an increase in SCN population density. An HG type test was conducted by SCN Diagnostics at the University of Missouri for the Georgetown field each season. HG type testing tests SCN reproduction growth on 7 indicator lines by using a calculated female index, which is determined by comparing the average number of females produced on the standard susceptible line to the number produced on the HG indicator type over a 30-day period (Niblack et al., 2002; Beeman et al., 2016). In addition to soil sampling, mid-season on-root female counts were quantified by destructively sampling five plants from the first row of each treatment. Plants were dug and roots were gently rinsed in field and brought back to the lab. Stems were clipped to separate root and above-ground plant parts. Roots were placed on an 850 μm mesh sieve over a 250 μm sieve and blasted with high pressure water for approximately 1 min to release females. The contents of the bottom sieve were rinsed and transferred to a counting dish where SCN females were enumerated under a dissecting microscope (Stemi 508, Carl Zeiss Microscopy LLC, Thornwood, NY). Female counts and fresh root weights were recorded and used to calculate the number of females per gram of root weight.

### Data Analyses

Mixed-model analysis of variance (ANOVA) was conducted using PROC GLIMMIX in SAS statistical software (SAS version 9.4, SAS Institute Inc. Cary, NC). Within both experiments, there were significant trial by run interactions, so runs within each experiment were analyzed individually for each year. Data were analyzed for percent emergence, SCN Rf, SCN female counts, females per gram of fresh root weight, and yield with seed treatment as a fixed effect, while replication and the overall error term were considered random effects. Both raw and transformed Rf values were used for presentation purposes. The Rf data were transformed using the equation log_10_ [(Pf+1)/(Pi+1)] prior to analysis to generate normal distribution values (Wang et al., 2000). Numerous SCN population studies have utilized log_10_ transformation to better represent reproduction in the field (Koenning et al., 1998; Brucker et al., 2005). The (x+1) equation was used to prevent the occurrence of a zero in the denominator for fields with low initial populations. Fixed effects were tested for significance at α = 0.05 and LSmeans were separated using paired *t* tests.

## Results

### Environmental Conditions

At the Georgetown site, both experiments of 2020 were planted on April 19, twenty-one days before the final frost on May 10. The average air temperature at planting was 9.1°C, while the average soil temperature was 11.1°C. The average temperature for the month of April was 11.2°C. Following planting, 42.1 ml of rain fell in 11 days, resulting in soil crusting that impeded soybean emergence. Emerged beans were slightly impacted by the late frost, but were able to outgrow the damage. The average total rainfall for the season, May through October, was 79.8 ml. Average monthly air temperatures during that period ranged from 15.5°C to 26.4°C. Average monthly soil temperatures ranged from 16.8°C to 28.1°C (DEOS, 2020).

In 2021, both experiments in Georgetown were planted on April 27 four days after the final frost event for the season. The average air temperature on the day of planting was 17.6°C and the soil temperature was 13.3°C. The average air temperature for April was 12.4°C. The total rainfall following planting was 13.6 ml in 3 days. The average total rainfall for the remainder of the growing season was 75.1 ml, May through October. The average monthly air temperature ranged from 17.1°C – 25.1°C. Average monthly soil temperatures ranged from 17.4°C to 26.7°C (DEOS, 2021).

#### Experiment 1

In 2020, rainfall totaling 42.1 ml in the week after planting led to crusting of the soil, making emergence for plants difficult (DEOS, 2020). Due to cold weather, no plants had emerged at 14 days after planting (DAP), so first counts were taken at 14 days after emergence (DAE) and again at 28 DAE. Significant treatment differences were observed among all three treatments at 14 DAE (Table 3). At 28 days after emergence, both seed treatments had higher percent emergence than the nontreated seed. At planting, baseline SCN populations ranged from 820 to 1000 J2 per 500 cc soil. At 30 days after emergence, J2 populations were lower and soil samples from plots with fluopyram seed treatment had larger nematode populations than soil samples from plots with pydiflumetofen and nontreated seed (*p* = 0.03). In plants sampled for root blasting, the highest level of SCN females (*p* = 0.03) were present on roots from nontreated (189) and pydiflumetofen-treated seed (179) compared to fluopyram-treated seed (54). Lowest females per gram of root (*p* = 0.01) were observed in plots with fluopyram-treated seed (Table 3). All three treatments in this experiment showed a decreasing J2 population density over the growing season, resulting in Rf values less than one and no significant differences among treatments (Table 3). Differences in yield were not observed (Table 3).

**Table 3.** Experiment 1 - Emergence, nematode Reproductive Factor (Rf) values, female counts, and yield data.

**Trial Information**	**% Emergence 14 DAP^z^**	**% Emergence 28 DAP**	**Rf^y^**	**Transformed Rf^x^**	**Root female counts**	**Females per gram of root**	**Yield(kg/ha)**	**Yield difference from nontreated seed (kg/ha)**
2020, Georgetown								
Nontreated	26.2c	54.0b	0.6	−0.33	189.0a	19.1a	2125.1	–
Saltro	54.3b	90.5a	0.2	−0.67	179.2a	23.9a	2313.4	+188.3
ILEVO	70.0a	86.4a	0.2	−0.83	53.8b	5.4b	2447.9	+322.8
*p*-value	0.0001	0.0001	*ns*	*ns*	0.03	0.01	*ns*	
2021, Georgetown								
Nontreated	53.3c	56.8c	7.6	0.19	531.6	125.8	2838	–
Saltro	82.4a	85.5a	3.6	0.75	473.4	94.8	3275.1	+437.1
ILEVO	68.9b	79.5b	6.6	0.69	482.2	115.3	3281.9	+443.9
*p*-value	<0.001	0.0001	*ns*	*ns*	*ns*	*ns*	*ns*	
2021, Queenstown								
Nontreated	53.3c	56.8b	76.17	0.74	1.6	0.1	4848.8	–
Saltro	82.5	85.5a	46.2	1.91	2.6	0.1	4815.2	−33.6
ILEVO	68.9a	79.5a	8.51	−0.06	0.8	0.05	4909.3	+60.5
*p*-value	<0.001	<0.0001	*ns*	*ns*	*ns*	*ns*	*ns*	

z Means followed by the same letter are not significantly different based on Fisher's Least Significant Difference (LSD; α = 0.05). *ns* = not significant.

y The reproductive factor (Rf) was calculated by dividing the final population density [Pf] by the initial population density [Pi] to generate a ratio to measure the change in number of SCN J2's per 500 cm^3^ of soil over the entire growing season for each plot. When Rf=1, no change in SCN population density occurred in the season; if Rf <1, there was a decrease in SCN population density over the season; if Rf >1, there was an increase in SCN population density.

x Reproductive factor data were transformed using the equation log_10_ [(Pf+1)/(Pi+1)] prior to analysis.

In 2021, in the same high-pressure SCN field in Georgetown, DE, pydiflumetofen-treated seed had the highest average percent emergence at 14 and 28 DAP (Table 3). Baseline SCN populations ranged from 10–140 J2 and increased over the season and Rf values greater than one. There were no differences in SCN soil or female counts among treatments and there were no differences in yield (Table 3).

At the second location with low SCN pressure in Queenstown, MD, treatment differences were observed in percent emergence 14 and 28 days after planting (Table 3). No treatment differences were observed in SCN populations at any of the sampling points. Initial SCN population values were low: in some cases, zero females were observed, resulting in large raw Rf values. Raw and transformed Rf values indicated an increase in population size over the season, but no treatment differences were observed. The average number of females per gram of root was less than one SCN female for all treatments (Table 3). Yields did not vary by treatment but were higher than the Georgetown location and ranged from 4815.2 kg/ha (71.6 bu/a) (pydiflumetofen) and 4909.3 kg/ha (73.0 bu/a) (fluopyram).

#### Experiment 2

Screening of HG types on SCN populations was conducted using a composite soil sample from Experiment 2 fields in 2020 and 2021. In 2020, the SCN population was designated as HG type 1.2, reproducing above 10% on indicator lines Peking (line 1; Female Index = 22%) and PI88788 (line 2; Female Index = 64.8%). The SCN populations for 2021 tested as HG type 2, capable of reproducing on PI88788 (line 2; Female Index = 66.2%).

In 2020, there were no differences in average emergence among treatments. No differences in soil egg counts were observed at any sample timing. SCN female counts on roots ranged from 380 (base treatment) to 201 (fluopyram-treated seed) with no differences among treatments (Table 4). No differences were observed in number of females per gram of roots. Initial nematode populations were high in 2020 and Rf values indicated declining population densities across all treatments over the season. No differences in yield were observed. Yield ranged from 2750.6 kg/ha (40.9 bu/a) in plots treated with *P. nishizawae* to 2037.7 kg/ha (30.3 bu/a) in plots with only the base treatment.

**Table 4.** Experiment 2 – Emergence, nematode Reproductive Factor (Rf) values, female counts, and yield.

**Trial Information**	**% Emergence 14 DAP^z^**	**% Emergence 28 DAP**	**Rf^y^**	**Transformed Rf^x^**	**Root female counts**	**Females per gram of root**	**Yieldkg/ha**	**Yield difference from base seed treatment (kg/ha)**
Georgetown, 2020								
Nontreated	74.6	na	0.5	−0.40	239	40.5	2353.8	+316.1
Fungicide + Insecticide Base	73.1	na	0.6	−0.29	380	57.7	2037.7	−
BioST	70.6	na	0.5	−0.39	323	46.8	2454.6	+416.9
Aveo + Base	75.4	na	0.7	−0.37	270.8	39.2	2528.6	+490.9
Clariva + Base	76.2	na	0.4	−0.40	290.7	37.8	2750.5	+712.8
ILEVO + Base	77.0	na	0.7	−0.25	200.8	34.6	2219.3	+181.6
Trunemco + Base	76.0	na	0.6	−0.29	292.0	43.9	2313.4	+257.7
Saltro + Base	77.7	na	0.6	−0.30	333.0	50.0	2084.8	+47.1
*p-value*	*ns*	na	*ns*	*ns*	*ns*	*ns*	*ns*	
Georgetown, 2021								
Nontreated	47.3c	48.51c	6.8	0.77	341.5	55.6	2885.1 d	−416.9
Fungicide + Insecticide Base	69.9ab	76.07ab	6.4	0.78	450.8	71.5	3302 b	−
BioST	69.2ab	74.4ab	8.4	0.84	392.7	52.1	3328.9 b	+26.9
Aveo + Base	70.3ab	78.8a	6.4	0.78	394.9	55.4	3059.9 c	−242.1
Clariva + Base	76.1a	78.7a	6.9	0.79	428.8	62.9	3080.1 c	−221.9
ILEVO + Base	68.0ab	74.0ab	8.0	0.82	202.8	36.5	3402.9 b	+100.9
Trunemco + Base	73.5ab	76.6ab	8.7	0.90	276.2	44.0	3295.3 b	−6.7
Saltro + Base	66.9b	72.5b	6.9	0.80	305.5	46.3	3813.1 a	+511.1
*p*-value	<0.0001	<0.0001	*ns*	*ns*	*ns*	*ns*	0.0005	

z Means followed by the same letter are not significantly different based on Fisher's Least Significant Difference (LSD; α = 0.05). ns = not significant.

y The reproductive factor (Rf) was calculated by dividing the final population density [Pf] by the initial population density [Pi] to generate a ratio to measure the change in number of SCN eggs per 250 cm^3^ of soil over the entire growing season for each plot. When Rf=1, no change in SCN population density occurred in the season; if Rf <1, there was a decrease in SCN population density over the season; if Rf >1, there was an increase in SCN population density.

x Reproductive factor data were transformed using the equation log_10_ [(Pf+1)/(Pi+1)] prior to analysis.

In 2021, emergence was lowest in plots with nontreated seed at both 14 and 28 DAP evaluations. The addition of a base treatment improved emergence over the nontreated seed and emergence of base-treated seed did not differ from treatments with an additional nematicide component. There were no significant differences between treatments for initial soil egg counts, end of season egg counts, 30 DAP SCN female root counts or the number of females per gram of roots. Soil samples in 2021 began with low population densities and Rf values indicated increasing SCN populations in the field over the season across all treatments (Table 4). Differences in yield were present (*p* < 0.001). Plots with pydiflumetofen-treated seed had the highest yield 3813.1 kg/ha (56.7 bu/a) and nontreated plots had the lowest 2885.1 kg/ha (42.9 bu/a).

## Discussion

SCN is a consistent concern to Mid-Atlantic soybean production due to widely prevalent loamy sand soils. In recent survey work across DE and MD, SCN was present in 53% of surveyed fields and above economic threshold for 66% of those samples (Kessler and Koehler, 2022). The widespread occurrence of this economically important pathogen has led to interest in the use of seed treatments as a management tool, but no data on local performance of nematode-protectant seed treatment products exists for the region. This is the first study to examine the use of pydiflumetofen as a seed treatment for management of SCN in DE and MD. This is also the first study examining biological products for SCN management in the region.

Across all trials, improved plant emergence was the most consistent effect of seed treatment. In this study nematode-protectant products were evaluated individually (Experiment 1) and in combination with base fungicide + insecticide treatments (Experiment 2). Nematode-protectant seed treatments can be chemical or biological. Within Experiment 1, both chemical treatments were SDHI fungicides also labeled for SCN management. In Experiment 2, base fungicides and insecticide treatments were layered with chemical and biological nematode-protectants. Percent emergence was not increased following the addition of any nematode protectant, indicating that the differences in percent emergence were likely due to the base fungicide component in Experiment 2 or the fungicide activity of pydiflumetofen and fluopyram compared to plain seed in Experiment 1.

Treatment differences for SCN J2 soil counts, female counts, and number of females per gram of root were observed in 2020, Experiment 1, where fluopyram-treated seed had the highest J2 soil count at 30 days after emergence, the lowest female counts at 30 DAE, and the lowest number of females per root at 30 DAE. Similar results were observed in a 2018 study where greenhouse trials with fluopyram-treated seed of susceptible and resistant varieties had reduced females per root and females per gram of root at 30 days after planting using diluted field soil (1000 eggs per 100 cm^3^) (Beeman and Tylka, 2018). The lack of replication in the remaining trials of Experiment 1 may be due to spatial variability (Avendaño et al., 2004) and changing population density in the soil (Beeman and Tylka, 2018) at the Georgetown site and low SCN pressure at the Queenstown site.

In 2020, nematode populations at the Georgetown field were highest at planting and declined through the season, demonstrated by low Rf values. During 2021 in the same field, however, initial populations were low and displayed increasing population sizes by harvest. Similar trends in plots with high sand content have been observed in previous work. In a previous study, plots with high Pi and low Pf values in the first year were followed by low Pi values and higher Pf values in year two. Changes in population were attributed to the environmental stress caused by sandy soils on plants and to host damage caused by the high SCN initial population density in year one of the trial (Koenning et al., 1988). Large initial population sizes likely damage the hosts to the point where large populations are no longer sustainable, thus resulting in population decline by harvest (Alston and Schmidt, 1987). Environmental conditions may also contribute to fluctuation in nematode populations. In Georgetown from December 2020 to March 2021, the average air temperature ranged from 2.3°C to 8.7°C while the average soil temperature ranged from 3.4°C to 8.7°C, approximately 2 to 3 degrees cooler than recorded air and soil temperature ranges from December 2019 to March 2020. The optimal temperature for SCN is 25°C and colder winter temperatures can reduce SCN populations (Niblack et al., 2005). It is possible the colder temperatures contributed to lower initial populations in 2021. It is also important to note that despite lower Pi values in Experiments 1 and 2 during 2021, Pf values in 2021 remained consistent with those from 2020, reinforcing the observation that changes in Pi do not always affect Pf results (Abawi and Jacobsen, 1984; Koenning et al., 1988). These findings support observations from other SCN field trials testing nematode seed treatment products where variability among location and environment were observed for Rf values (Bissonnette et al., 2018, 2020; Kandel et al., 2017, 2019).

Treatment differences in Rf were not observed in any year of either experiment. This is consistent with a field and greenhouse study in Michigan where fluopyram was used to suppress SCN population densities, though none of the recorded Rf values were significant (Roth et al., 2020). This is also consistent with results from Iowa, in which fluopyram-treated seed increased yields but did not significantly decrease SCN Rf values across 12 strip trials and 27 small plots (Bissonnette et al., 2020). Results from Bissonnette et al. 2020 differed from previous work where nematicide seed treatments, with the addition of a fungicide and insecticide base, were able to reduce some SCN reproduction, but not increase yield (Gaspar et al., 2014; Mourtzinis et al., 2017; Bissonnette et al., 2018). In the current study, fields with higher Pi values averaged lower Rf values. Effects of initial population density on Rf have been noted previously (Alston and Schmidt, 1987; Roth et al., 2020). When fields begin with low initial SCN populations, any increase will result in larger Rf values, which may make Rf a more useful indicator for low population environments. The Rf values for the Queenstown location are an example where low initial populations resulted in numeric spread of Rf values. In field sites like Georgetown 2020 where initial populations were high, Pf combined with Rf may be a better indicator of in-season changes (Roth et al., 2020).

Yield differences were observed in Experiment 2 in 2021 where plots with pydiflumetofen-treated seed had the highest yield and plots with nontreated seed had the lowest yield. Numerical differences of yield in Experiment 2 plots with nematicide seed treatments compared to plots with only base-treated seed ranged from a 242.1 kg/ha (3.6 bu/a) reduction to a 712.9 kg/ha (10.6 bu/a) increase (Table 4). The 2021 season began with lower baseline SCN populations; environmental conditions were favorable during pod set and fill, likely resulting in less plant stress and higher overall yields than 2020. Previous studies have shown that the inclusion of a nematicide with an insecticide + fungicide base does not necessarily result in a significant increase in yield (Mourtizinis et al., 2017). Stand emergence was the most consistent response of seed treatment across both experiments. It is likely that improved emergence was a factor in positive yield trends observed (Tables 3 and 4), but the ability of soybean plants to bush under reduced populations kept yield differences lower than may have been anticipated based on emergence percentages. Yield responses in this trial are reflective of previous work examining the effects of fluopyram on SCN reproduction and soybean yield, in which fluopyram improved yields compared to a control by 52.2 to 248.8 kg/ha in small plots and strip trials (Bissonnette et al., 2020).

Foliar symptoms of Soybean Sudden Death Syndrome (SDS) caused by *F. virguliforme* were not present in any of the field sites during 2020–2021, despite the presence of SCN, early planting for the region, and the presence of SDS within the region (Kessler and Koehler, 2022). Previous studies have shown that in the absence of SDS, fewer yield differences may be observed among treatments, and differences in SCN reproduction and Pf may not be present when comparing seed treatments to base or nontreated seed (Kandel et al., 2017). The interaction between SDS and SCN limits the ability to study seed treatment effects on either SDS or SCN (Bissonnette et al., 2020). Many of the field studies testing fluopyram have focused on root and foliar symptoms caused by *F. virguliforme* colonization, with less focus on SCN (Kandel et al., 2017; Roth et al., 2020; Sjarpe et al., 2020; Wang et al., 2019). To separate SDS and nematicide activity in the field, SDS disease index levels are recommended to be below 10 to determine if yield treatment effects are related to SCN management (Kandel et al., 2017). *F. virguliforme* was not recovered in soil tests and these experiments provide assessment of seed treatments for SCN control in the absence of SDS pressure.

Results of Hg tests in this trial were consistent with previous Mid-Atlantic surveys (Mulrooney and Gregory, 2010), with reproduction levels of 64.8% and 66.2% on PI88788 lines in 2020 and 2021, respectively. While Hg type 2 was expected, the recovery of Hg 1.2 in 2020 indicated elevated reproduction on Peking lines as well. The Peking resistance source has been available since 1957 (Ross and Brim, 1957), but to date, there have been few commercially available soybean varieties containing Peking resistance in soybean maturity groups conducive for the region. Although this has limited the use of Peking resistance in the Mid-Atlantic, nematode reproduction rates should still be monitored in fields utilizing Peking as a resistance source.

Four of the treatments in Experiment 2 were biological materials including gram-positive *Bacillus* products (Trunmeco™ Aveo™EZ) and *Pasteuria* (Clariva®pn) and gram-negative heat-killed *Burkholderia* (BioST^®^Nematicide 100) (Table 2). Biological seed treatments have mixed efficacy for nematode management (Musil, 2016; Abdel-Salam et al., 2018; Ghahremani et al., 2020). There are several contributing factors, but one of the most important is viability of soil environments (Lamovšek et al., 2013). Soil environments that inhibit establishment of the biological components reduce or nullify the effects of the seed treatment, resulting in little to no nematode control. In Wisconsin field trials no consistent SCN reductions or yield benefits were observed when using seed treated with *P. nishizawae* (Lund et al., 2018). This finding was consistent with other work showing the density of endospores may impact the efficacy of SCN parasitism (Bissonnette et al., 2018). In Iowa field trials, *P. nishizawae* was shown to significantly decrease Rf values of SCN in soybeans and improve yield when compared to CruisserMaxx Advanced plus Vibrance (Bissonnette et al., 2018). In 2020, the *P. nishizawae* treatment had the highest numerical yield in Experiment 2. Aerial drone imagery identified that four replications of the plots with *P. nishizawae*–treated seeds were randomly located in the top-yielding portion of the field. This likely contributed to the higher yield observed. In 2021, the *P. nishizawae* treatment was numerically lower than the base fungicide + insecticide seed treatment (−221.9 kg/ha), indicating that *P. nishizawae* yield response may be variable and dependent on other biotic and abiotic conditions.

While the effect of pydiflumetofen on egg, cyst, and juvenile reduction has been explored in greenhouse soybeans (Dhital, 2020), evaluation of efficacy in a field setting is limited due to its recent commercial release and first field use in 2020. The most notable effect of seed treatments was improved stand emergence over nontreated seed, but differences in percent emergence of base-treated seed did not differ following the addition of any nematicide to the base. As observed in previous studies, performance of nematode seed treatment products can be inconsistent across field experiments (Bissonnette et al., 2018; Roth et al., 2020). Nematode response varied across trials and by year. In 2020, nematode populations were reduced in plots with fluopyram-treated seed in one trial. In 2021, yields of pydiflumetofen-treated plots were higher than all other treatments in one experiment. Seed treatments do not offer a stand-alone solution to nematode control but may be useful to the region as an additional management tool incorporated as part of an integrated management approach for SCN control. Future work could continue to investigate the efficacy of nematode seed treatment products in combination with emerging SCN resistance sources, or in the context of cultural approaches such as use of cover crops and/or host rotation.
